# Non-optimum temperature increases risk and burden of acute myocardial infarction onset: A nationwide case-crossover study at hourly level in 324 Chinese cities

**DOI:** 10.1016/j.eclinm.2022.101501

**Published:** 2022-06-17

**Authors:** Yixuan Jiang, Jialu Hu, Li Peng, Huichu Li, John S. Ji, Weiyi Fang, Hongbing Yan, Jiyan Chen, Weimin Wang, Dingcheng Xiang, Xi Su, Bo Yu, Yan Wang, Yawei Xu, Lefeng Wang, Chunjie Li, Yundai Chen, Dong Zhao, Haidong Kan, Junbo Ge, Yong Huo, Renjie Chen

**Affiliations:** a School of Public Health, Key Lab of Public Health Safety of the Ministry of Education and NHC Key Lab of Health Technology Assessment, Fudan University, Shanghai, China; b Department of Cardiology, Zhongshan Hospital, Fudan University, Shanghai Institute of Cardiovascular Diseases, Shanghai, China; c Shanghai Key Laboratory of Meteorology and Health, Shanghai Meteorological Bureau, Shanghai, China; d Department of Environmental Health, Harvard T.H. Chan School of Public Health, Boston, MA, USA; e Vanke School of Public Health, Tsinghua University, Beijing, China; f Department of Cardiology, Huadong Hospital Affiliated to Fudan University, Shanghai, China; g Center for Coronary Artery Diseases, Chinese Academy of Medical Sciences in Shenzhen, Shenzhen, China; h Center for Coronary Artery Diseases, Chinese Academy of Medical Sciences, Beijing, China; i Department of Cardiology, Guangdong Provincial People's Hospital, Guangzhou, China; j Department of Cardiology, Peking University People's Hospital, Beijing, China; k Department of Cardiology, General Hospital of Southern Theater Command, Guangzhou, China; l Department of Cardiology, Wuhan ASIA General Hospital, Wuhan, China; m Department of Cardiology, The 2nd Affiliated Hospital of Harbin Medical University, Harbin, China; n The Key Laboratory of Myocardial Ischemia, Chinese Ministry of Education, Harbin, China; o Department of Cardiology, Xiamen Cardiovascular Hospital Xiamen University, Xiamen, China; p Department of Cardiology, Shanghai Tenth People's Hospital, Shanghai, China; q Heart Center and Beijing Key Laboratory of Hypertension, Beijing Chaoyang Hospital, Capital Medical University, Beijing, China; r Department of Emergency, Tianjin Chest Hospital, Tianjin, China; s Department of Cardiology, Chinese PLA General Hospital, Beijing, China; t Department of Epidemiology, Beijing An Zhen Hospital, Capital Medical University, Beijing Institute of Heart, Lung and Blood Vessel Diseases, Beijing, China; u IRDR ICoE on Risk Interconnectivity and Governance on Weather/Climate Extremes Impact and Public Health, Fudan University, Shanghai, China; v Department of Cardiology, Peking University First Hospital, Beijing, China

**Keywords:** Ambient temperature, Acute myocardial infarction, Case-crossover study, Attributable fraction, Centralized heating

## Abstract

**Background:**

The associations of ambient temperature with acute myocardial infarction (AMI) have seldom been examined based on the time of symptom onset.

**Methods:**

We conducted a time-stratified case-crossover study among 1,046,773 eligible AMI patients from 2,093 hospitals in 324 Chinese cities from January 1, 2015 to June 30, 2021, after excluding those transferred from other hospitals or having not reported the time of symptom onset. Hourly exposure to ambient temperature was calculated as multiple moving 24-h averages (days) before hourly onset of AMI symptoms. Conditional logistic regression and distributed lag non-linear models with a duration of 0–21 days were used to estimate the cumulative associations of non-optimum temperature with AMI onset and the corresponding disease burden nationally. Subgroup analyses by region and period were conducted. Specifically, cities with and without centralized heating system were classified into heating and non-heating regions, respectively. The whole year in heating region was divided into heating and non-heating periods based on the duration of centralized heating in each city.

**Findings:**

Almost monotonically increasing risks were observed for both overall AMI and its two subtypes when ambient temperature declined. The effects of extremely low temperature occurred immediately on the concurrent day, and lasted up to almost 3 weeks. The excess risks of AMI onset associated with non-optimum ambient temperatures were observed during the whole year in the non-heating region and non-heating period in the heating region, but not during heating period. Specifically, odds ratios of AMI onset associated with extremely low temperature cumulated over 0–21 days were 1.24 (95% CI: 1.13–1.37), 1.46 (95% CI: 1.20–1.76), and 1.62 (95% CI: 1.46–1.81) in the heating region during non-heating period, in the non-heating region during winter and non-winter period, respectively. The heat effects on AMI onset were very modest and transient. Totally, 13.26% of AMI cases could be attributable to non-optimum temperatures nationally. The burden of AMI attributable to non-optimum temperature was much smaller in heating region than in non-heating region. Somewhat stronger effects were observed in females and patients aged older than 65.

**Interpretation:**

This nationwide study provided robust evidence that non-optimum ambient temperature may significantly trigger AMI onset, and for the first time estimated the disease burden after accounting for spatial and seasonal heterogeneity. Centralized heating might substantially mitigate AMI burden due to non-optimum temperature.

**Funding:**

Shanghai International Science and Technology Partnership Project, National Natural Science Foundation of China, Talent Training Program of Zhongshan Hospital, Fudan University.

**Research in context**

**Evidence before this study**

We searched PubMed, Web of Science, and Embase up to February 25, 2022, using search terms “ambient temperature”, “acute myocardial infarction”, “ischemic heart disease”, “disease burden”, and “attributable” without language restrictions, to identify papers on the relationships between non-optimum ambient temperature and acute myocardial infarction (AMI). Most studies fitted aggregate cases of AMI-related death or hospitalization with exposures at the daily timescale through time-series design, which could be challenged by ecological fallacy and unclear chronological order of exposure and events within the same day. Researches quantifying the temperature-related risk and burden were mainly conducted in individual cities or small areas, and relied on death which may be not as sensitive and immediate as disease onset. Thus, a systematic investigation on the associations of non-optimum temperature with AMI onset at the individual level, and a careful spatiotemporal evaluation on AMI burden attributable to non-optimum temperature are needed.

**Added value of this study**

In this nationally-representative case-crossover study of 1.05 million AMI cases from 2,093 hospitals in 324 Chinese cities, we found that non-optimum ambient temperature may significantly trigger AMI onset and contribute a considerable burden. The associations of extremely low temperature occurred immediately on the concurrent day of exposure, and lasted up to almost 3 weeks, whereas the heat effects on AMI onset were very modest and transient. No excess risk was observed during heating period in the region with centralized heating system, while the region without centralized heating showed much larger excess risk and burden.

**Implications of all the available evidence**

Tailored public health interventions (e.g., early warning system) and resilient infrastructures (e.g., centralized heating) are needed to reduce the hazardous cardiovascular effects of non-optimum ambient temperature under the context of global climate change. Spatial and seasonal heterogeneity should be taken into consideration to make a more accurate evaluation on the risk and burden associated with non-optimum ambient temperature.

Alt-text: Unlabelled box

## Introduction

Ischemic heart disease (IHD), especially acute myocardial infarction (AMI), is a leading cause of disease burden that contributes to approximately 9 million premature deaths and 182 million disability-adjusted life-years (DALYs) worldwide in 2019.^1^^,^^2^ The non-optimum ambient temperature was listed as an independent environmental risk factor for the first time in the Global Burden of Disease (GBD) Study 2019 and was estimated to account for 597 thousand deaths and 11 million DALYs of IHD globally.^3^^,^^4^ However, this evaluation failed to take lagged effects of temperature into consideration,^3^ which is important to accurately quantify the risk and burden of disease due to non-optimum temperature exposure.^5^ Further, previous findings on the effects of ambient temperature on IHD or AMI were obtained mainly from time-series studies that considered daily average as the metric of exposure and daily aggregate counts of IHD or AMI hospitalization or death as the outcome. Thus, the results could be prone to ecological fallacy and unclear chronological order of exposure and events within the same day.^5^, ^6^, ^7^ Besides, few studies have compared the associations between two AMI subtypes AMI (i.e., ST-segment-elevation myocardial infarction, STEMI, and non-ST-segment-elevation myocardial infarction, NSTEMI). Consequently, an individual-level case-crossover study based on hourly onset data of AMI as well as its two subtypes could provide robust support for more accurate estimation of the relative risk and disease burden due to non-optimal temperature. However, such kind of studies was rarely conducted. It also remains unclear on the corresponding disease burden estimated according to the results of case-crossover studies at hourly level.

Cold typically constitutes the disproportionately high proportion of disease burden due to non-optimum temperatures,^3^ and its health risks and disease burden could be empirically mitigated by adequate air conditioning or heating. There has been a long history of indoor heating in cities of cool or cold climate worldwide, which could drastically alter the indoor temperature, despite different outdoor temperatures. In China, the city centralized-heating policy was created since the 1950s in the north of a line along the Qin Mountains and the Huai River, known as the *Qin-Huai line*. Typically, cities north of the line have centrally controlled public heating system (i.e., centralized heating system), which usually comes online in November and lasts to March or April with on-off adjusted to regions and specific temperature of that year. In contrast, those south of the line have no centralized heating. Still, little empirical evidence was available about the possible effect modifications by the policy of centralized heating in AMI risk and burden due to non-optimum temperatures.

In this study, we conducted a time-stratified case-crossover analysis using a nationwide registry database of China to examine the associations between non-optimum temperature and hourly onset of AMI and its two subtypes. We further estimated the burden of AMI onset that could be attributable to non-optimum temperature nationally and by region and period classified by the presence of centralized heating.

## Methods

### Study population and outcome data

Data of AMI onset was extracted from the Chinese Cardiovascular Association (CCA) Database-Chest Pain Center. Established in 2015, this database is a nationally representative and multicenter registry.^8^ Patients with acute chest pain visiting the chest pain center are reported to the registry. Information on individual characteristics (e.g., sex and age), date and time of symptom onset, diagnosis, results of clinical examinations and treatment procedures were collected and inputted into the registry database according to their medical records by well-trained staffs in each center.^9^ A web-based information system with automatic data check was developed to avoid invalid and illogical values.

For this analysis, we retrieved data on AMI patients that were recorded in the database between January 1, 2015 and June 30, 2021. Patients who were transferred from another hospital after the symptom onset or who did not report the time of symptom onset were excluded. All AMI diagnoses were made by cardiologists and were based on symptoms, electrocardiography, and laboratory test results (e.g., cardiac troponin levels), according to the Chinese Society of Cardiology guidelines.^10^, ^11^, ^12^ All AMI cases were further classified into STEMI and NSTEMI based on their electrocardiography results.

The Institutional Review Board at the School of Public Health, Fudan University approved the study protocol (IRB#2021-04-0889), and waived the requirement for informed consent because this analysis only involved deidentified data and no patients could be contacted. This study was presented according to the guidelines of Strengthening the Reporting of Observational Studies in Epidemiology (STROBE).

### Study design

We used a time-stratified case-crossover design in this study. This case-only design allows each case to serve as his/her own control, and therefore, factors that are time-invariant or are stable within a short period of time (e.g., demographic, socioeconomic, and behavioral risk factors) are automatically controlled.^13^^,^^14^ For this analysis, the case index hour was defined as the hour of self-reported AMI symptom onset. Correspondingly, we matched each case index hour with 3 or 4 control index hours in the same year, month, day of the week, and hour of day to control for long- and mid-term variations and seasonality. To control for possible time trends, we selected control hours both before and after the case hour within the same month. For example, if the first AMI symptom occurred at 10 AM on Wednesday, June 20, 2018, we would define 10 AM on Wednesday, June 20, 2018 as the case index hour and 10 AM on all other Wednesdays in June 2018 (i.e., June 6, 13, and 27) as the control index hours.

### Exposure data

Hourly temperature and relative humidity data were obtained from weather stations in the China Meteorological Data Service Center (http://data.cma.cn/). To reduce the impact of outliers, we excluded temperatures exceeding 3 times standard deviation before statistical analyses nationally and by region and period, respectively. Because approximately 55% of the patients did not report the complete address of their location at the time of disease onset, we matched environmental exposure data from the nearest weather station to the address of reporting hospital. To reduce exposure measurement error, we only included hospitals that were within 100 km from the nearest weather station. We further collected hourly concentrations of criteria pollutants (i.e., fine particulate matter, nitrogen dioxide, sulfur dioxide, ozone and carbon monoxide) from the National Urban Air Quality Real-time Publishing Platform (http://106.37.208.233:20035/) for the adjustment of air pollution. To reduce exposure misclassification, we only assigned pollutants data to hospitals that were within 50 km from the nearest fixed-site air quality monitors.

### Statistical analysis

#### Associations of temperature and AMI onset

We used conditional logistic regression models combined with distributed lag non-linear models (DLNM) to explore possible non-linear exposure–response relationships over different lag periods (Supplemental Methods). Briefly, for each case or control, we calculated a set of 24-h moving averages (days) before the index hour for up to 21 days prior. We utilized daily lags rather than hourly lags in order to reduce the enormously huge computation burden, as well as to facilitate the risk communication and comparability with most previous studies.^15^, ^16^, ^17^, ^18^ We selected a maximum of 21 lag days *a priori* as many previous studies reported that short-term effects of low temperature on cardiovascular disease could last for about 3 weeks.^13^^,^^16^^,^^18^ First, we used DLNM to construct the cross-basis function for temperature. A natural cubic spline function (degrees of freedom, *df*= 4) with three internal knots at equally spaced percentiles (25^th^, 50^th^ and 75^th^) of temperature ranges was fitted to account for potential non-linear exposure–response relationships. A natural cubic spline function (*df*= 4) with two internal knots at equally spaced log-values of lags (0–21 d) was applied to allow for more flexibility at shorter delays. These parameters were empirically determined according to previous literatures.^15^^,^^19^^,^^20^ Then, we included this cross-basis function in a conditional logistic regression model to estimate the association between ambient temperature and AMI onset, adjusting for a binary indicator of public holidays and a natural cubic spline with 3 *df* for relative humidity (0–21 days average prior to the case or control hour). The odds ratios (ORs) and 95% confidence intervals (CIs) of AMI were derived from the conditional logistic models by comparing a given temperature to the referent temperature with the lowest AMI risk. We further calculated ORs and 95%CIs of AMI by comparing the extremely low (i.e., the 1^st^ percentile of the temperature distribution) and high temperatures (the 99^th^ percentile of the temperature distribution) to the referent, respectively.^21^^,^^22^ To observe possibly differential associations with AMI subtypes, we fitted separate models for overall AMI, STEMI, and NSTEMI, respectively.

Considering the potential effect modifications by centralized heating, we further fitted the aforementioned main models separately in different regions and periods. Specifically, cities with and without centralized heating system were classified into heating and non-heating regions, respectively. The whole year in heating region was divided into heating and non-heating periods based on the duration of centralized heating, which was collected from local governmental documents in each city. As a comparison, the whole year was divided into the winter (December to February in the next year) and non-winter period in the non-heating region.

In addition, we fitted separate models in subgroups of sex (male vs. female) and age (< 65 vs. ≥ 65 years). Stratum-specific estimates were compared using the following formula:

(1) $$z=\frac{\beta_{1}-\beta_{2}}{\sqrt{{\text{SE}}_{1}^{2}+{\text{SE}}_{2}^{2}}}$$

where $\beta_{1}$ and $\beta_{2}$ are the stratum-specific regression coefficients (ln OR), and $SE_{1}$ and $SE_{2}$ are the corresponding standard errors.^14^

Prior evidence has suggested that the associations of high temperature with cardiovascular diseases appeared very immediately and were occasionally accompanied by a displacement of mortality or morbidity (the so-called harvesting effect).^20^^,^^23^ Thus, we additionally conducted an exploratory analysis to assess the potential associations between heat and AMI onset using hourly temperatures for up to 72 h.

We conducted several sensitivity analyses. First, we additionally adjusted for criteria air pollutants one by one and all pollutants at once in the main model to control for potential confounding by using the 0–24 h average concentrations before the index hour. Second, we restricted the analysis to participants who provided complete address of their location at the time of AMI onset and then compared estimates derived using temperatures measured near these locations to those measured near the hospital address. Third, we further limited the analysis to patients whose location of AMI onset was within 50 and 20 km from the nearest weather station, respectively, to explore the possible influence of different magnitude of exposure misclassifications. Fourth, we matched each case and control with regular daily mean temperature, and reran the main model to test whether using traditional daily average temperature would change the results. Fifth, we applied different *df* for temperature (*df*= 4–7; i.e., 3–6 knots), *df* for lag dimensions (*df*= 4–7; i.e., 2–5 knots) and the maximum of lag (i.e., 14 and 28 days) in DLNM to test the robustness of results. Last, we further applied a two-stage analytical framework, in which city-specific associations were estimated using the above-mentioned first-stage models and regional or national average associations were obtained by using the random-effects meta-analysis in the second stage to account for potential heterogeneity across cities.

#### AMI fractions attributable to non-optimum temperature

We applied the backward method proposed in a previous study to quantify the burden of AMI onset due to non-optimum temperatures,^24^ which could summarize the current-day burden related to exposures in the past 21 days. This approach accounts for the potentially complex temporal patterns of exposure–response relationships to provide more appropriate estimates for the disease burden, and thus has been widely used in previous studies within the framework of DLNM.^7^^,^^17^^,^^22^^,^^25^ The formulas of backward attributable fraction (AF) and number (AN) at time *t* were as follows^24^:

(2) $$AF_{x,t}=1-e^{\left(-\sum_{l=l_{0}}^{L}\beta_{x_{t-l},l}\right)}$$

(3) $$AN_{x,t}=AF_{x,t}\times N_{t}$$

$N_{t}$ denotes the number of cases at time *t*, while $AN_{x,t}$ and $AF_{x,t}$ indicate the number of cases and the related fractions at time *t* attributable to past exposures to x in the period *t − l_0_*, …, *t − L*, compared to the referent exposure. Given the potential regional and seasonal heterogeneity, we first calculated the ANs in each city based on region- and season- specific associations. Temperature with the lowest incidence risk of AMI was used as the common referent for calculating the season-specific disease burden for a given city. Then, the region- and season- specific ANs were computed by aggregating ANs of all cities in the corresponding region and season. Finally, the ratio of AN to the total number of cases in the corresponding region and season was yielded as regional and seasonal AF. Similarly, the national AF was calculated by dividing the sum of ANs across all cities by the national total number of AMI cases. Besides, empirical confidence intervals (eCIs) were calculated using Monte Carlo simulations by assuming a multivariate normal distribution of point estimate and (co)variance matrix derived from the regression model.^24^ The corresponding 2.5^th^ and 97.5^th^ percentiles of the distributions by simulating 5000 random samples were interpreted as the 95% eCIs.^22^^,^^24^^,^^25^

All analyses were conducted in R (Version 4.0.0, R Project for Statistical Computing) with the “*dlnm*” and “*survival*” packages for fitting main model, and the “*mvmeta*” package for performing the meta-analysis. The AF was calculated by function “*attrdl”* provided by Gasparrini et al.^24^ All tests were two-sided with an alpha of 0.05.

### Role of the funding source

The funding sources had no role in the design and conduct of the study; collection, management, analysis, and interpretation of the data; preparation, review, or approval of the manuscript; and decision to submit the manuscript for publication. The corresponding authors had full access to all data in the study and had the final responsibility to submit it for publication.

## Results

### Descriptive results

A total of 1,394,704 AMI patients were initially recorded in the CCA Database-Chest Pain Center from January 1, 2015 to June 30, 2021. After exclusion, there were 1,046,773 patients from 2,093 certified hospitals in 324 cities eligible for the final analysis (Figure S1 and Figure S2). Demographic characteristics were similar among all AMI patients and patients included in the final analysis (Table S1). Among the included AMI patients, 49.1% were aged over 65, 73.4% were male, 63.4% had STEMI, and 52.0% were from the centralized-heating region (Table S2). Average temperature and humidity over 0–24 h before the index hour during the study period were 15.1°C and 66.9%, respectively.

### Associations between temperature and AMI onset

**Figure 1:**
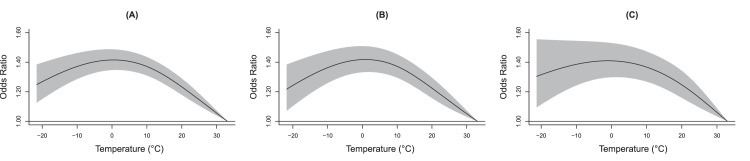
**Cumulative exposure–response curves for the associations of temperature with AMI (A), STEMI (B) and NSTEMI (C) onset across 324 cities over 0–21 days.** The solid black lines are the odds ratios of AMI onset, and the gray areas are the 95% confidence intervals. Abbreviations: AMI, acute myocardial infarction; STEMI, ST-segment-elevation myocardial infarction; NSTEMI, non-ST-segment-elevation myocardial infarction.

As shown in Figure 1, the highest temperature (33.1°C) had the lowest risk and thus was used as the referent for nationwide analysis. At the national level, we observed increasing risks of AMI and its two subtypes when ambient temperature declined, accompanied by a plateauing and even decreasing phenomenon at low temperatures.

**Figure 2:**
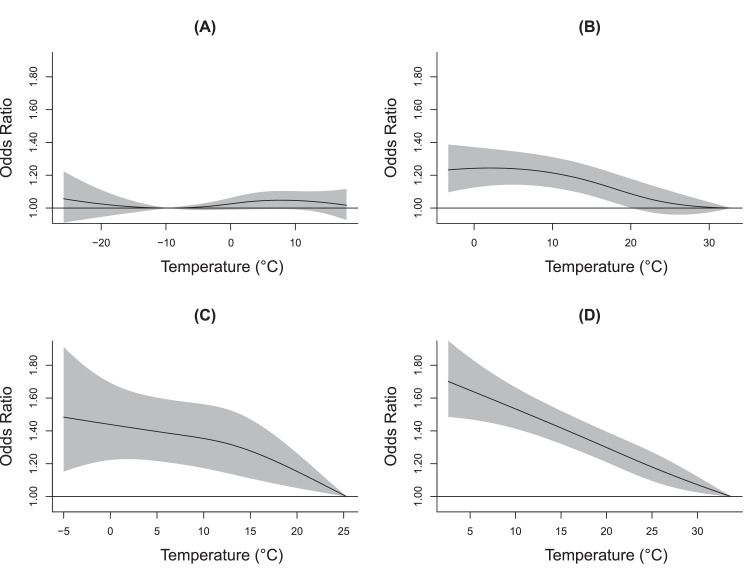
**Cumulative exposure–response curves for temperature with AMI onset over 0–21 days by region and period.** (A) heating region during heating period; (B) heating region during non-heating period; (C) non-heating region during winter; (D) non-heating region during non-winter period. The solid black lines are the odds ratios of AMI onset, and the gray areas are the 95% confidence intervals. Abbreviations: AMI, acute myocardial infarction.

**Figure 3:**
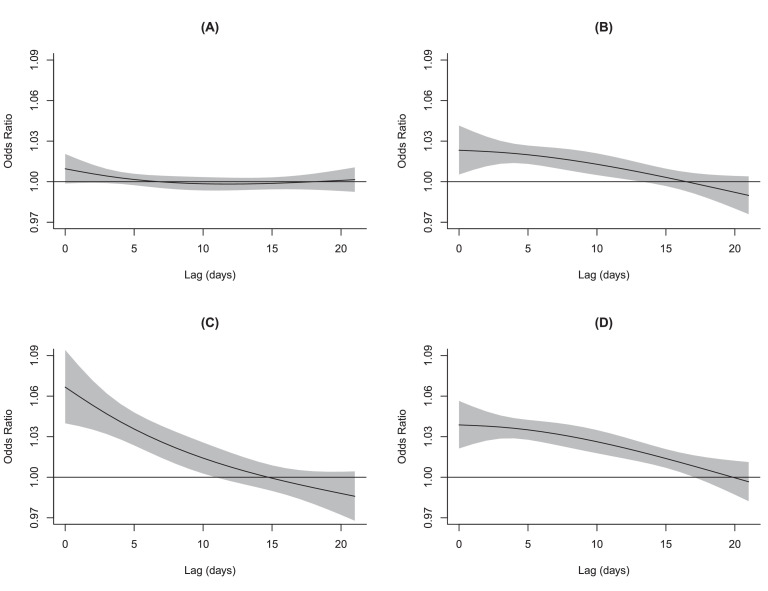
**Lag structures for the associations of AMI onset with extremely low temperature by region and period.** (A) heating region during heating period; (B) heating region during non-heating period; (C) non-heating region during winter; (D) non-heating region during non-winter period. The solid black lines are the odds ratios (extremely low temperature: 1^st^ percentile of temperature vs. the minimum risk temperature) of AMI onset, and the gray areas are the 95% confidence intervals. Abbreviations: AMI, acute myocardial infarction.

There were varying exposure–response relationships and lag patterns by regions and periods (Figure 2, Figure 3, and Table 1). In the heating region, null associations were found between ambient temperature and AMI during the heating period (Figure 2A and Figure 3A); however, the exposure–response curve during non-heating period (Figure 2B) was almost monotonically increasing but followed by a levelling-off at extremely low temperature (0.6°C) with the OR equaling to 1.24 (95% CI: 1.13–1.37) compared to the referent (32.8°C). In the non-heating region, monotonically increasing curves were also illustrated during both winter (Figure 2C) and non-winter period (Figure 2D); the corresponding ORs were 1.46 (95% CI: 1.20–1.76) and 1.62 (95% CI: 1.46–1.81) associated with extremely low temperature (-2.0°C and 6.0°C), relative to the respective referent temperatures (25.3°C and 33.7°C). As depicted in Figure 3, significant risk of AMI onset associated with extremely low temperature appeared on the concurrent day of exposure. Thereafter, the associations gradually attenuated and became statistically non-significant at approximately lag 13 days during the non-heating period in the heating region, 10 days during winter and 17 days during non-winter period in the non-heating region. Similar exposure–response curves and lag patterns were observed for STEMI and NSTEMI, except that the associations for NSTEMI during the non-heating period in the heating region became significant at lag 2 days or later (Figure S3 and Figure S4).

**Table 1:** Cumulative odds ratios (95%CIs) of AMI onset associated with extremely low temperature by region and period*^a^*.

Region	Extremely low temperature (°C)	Referent temperature (°C)	AMI	STEMI	NSTEMI
National	-14.2	33.1	1.33 (1.24, 1.44)	1.32 (1.20, 1.45)	1.36 (1.20, 1.55)
HH	-19.4	-9.6	1.02 (0.95, 1.10)	1.02 (0.94, 1.10)	1.04 (0.89, 1.22)
HNH	0.6	32.8	1.24 (1.13, 1.37)	1.24 (1.10, 1.39)	1.25 (1.06, 1.48)
NW	-2.0	25.3	1.46 (1.20, 1.76)	1.31 (1.02, 1.67)	1.74 (1.29, 2.35)
NNW	6.0	33.7	1.62 (1.46, 1.81)	1.55 (1.35, 1.78)	1.74 (1.48, 2.06)

Note: *^a^* extremely low temperature refers to the 1^st^ percentile of temperature, and the referent temperature is the minimum risk temperature.

Abbreviations: CIs, confidence intervals; AMI, acute myocardial infarction; STEMI, ST-segment-elevation myocardial infarction; NSTEMI, non-ST-segment-elevation myocardial infarction; HH, heating region during heating period; HNH, heating region during non-heating period; NW, non-heating region during winter; NNW, non-heating region during non-winter period.

Using hourly lags up to 72 h, our exploratory analyses demonstrated that high temperatures could transiently increase the risk of AMI onset within 20 h after exposure in the heating region during non-heating period (Figures S5–S9). However, significant reductions in risk were observed at longer lags, suggesting a harvesting effect for heat-related risk of AMI. The cumulative effects of high temperatures over 0–72 h were not significant (Table S3).

Significantly higher risks of AMI onset associated with extremely low temperature were found among females (OR: 1.52, 95%CI: 1.31–1.75) than males (OR: 1.26, 95%CI: 1.16–1.38). Moreover, the associations were somewhat stronger among the elders (OR: 1.41, 95%CI: 1.26–1.57) than their younger counterparts (OR: 1.25, 95%CI: 1.13–1.39); however, the between-group difference was non-significant (Table S4).

Our sensitivity analyses revealed robust associations after adjusting for air pollutants (Table 2). ORs were similar when using temperature measured near the location of AMI onset (OR: 1.50, 95%CI: 1.33–1.68) compared to the estimates using temperature measured near the hospital address (OR: 1.49, 95%CI: 1.32–1.67). Results remained stable when the analysis was further restricted to patients whose location of AMI onset was within 50 and 20 km to the nearest weather station, respectively (Table 2). Using traditional daily mean temperature did not appreciably change our results except that the associations became slightly weaker in the non-heating region during winter (Figure S10, Figure S11, and Table S5). Different combination of *df* for temperature and lag dimensions in DLNM generally yielded similar effect estimates (Table S6). When using a maximum lag of 14 days, the associations were weaker in the non-heating region during non-winter period, indicating that this lag structure cannot fully capture the effects of low temperature. The results were broadly similar when using a maximum lag of 28 days (Table S7). At last, the regional- or national- risk estimates also remained stable when using a two-stage meta-analysis (Table S8).

**Table 2:** Estimated odds ratios (95%CIs) of AMI onset associated with extremely low temperature^a^ over 0–21 days adjusting for pollutants among cases with pollutants data, and among cases with complete address of AMI onset.

Models	N	AMI	STEMI	NSTEMI
Main analysis	1,046,773	1.33 (1.24, 1.44)	1.32 (1.20, 1.45)	1.36 (1.20, 1.55)
Analysis among cases with pollutants data				
Main results b	893,675	1.32 (1.22, 1.43)	1.31 (1.19, 1.45)	1.33 (1.16, 1.53)
+ PM_2.5_	893,675	1.33 (1.23, 1.45)	1.34 (1.21, 1.49)	1.32 (1.14, 1.52)
+ NO_2_	893,675	1.29 (1.19, 1.41)	1.30 (1.17, 1.45)	1.27 (1.10, 1.47)
+ SO_2_	893,675	1.32 (1.21, 1.44)	1.33 (1.20, 1.48)	1.30 (1.13, 1.50)
+ O_3_	893,675	1.35 (1.24, 1.47)	1.36 (1.23, 1.51)	1.34 (1.16, 1.55)
+ CO	893,675	1.33 (1.22, 1.44)	1.33 (1.20, 1.48)	1.31 (1.14, 1.52)
+ all five pollutants	893,675	1.31 (1.20, 1.42)	1.32 (1.19, 1.47)	1.28 (1.11, 1.48)
Hospital-address analysis c	479,372	1.49 (1.32, 1.67)	1.51 (1.30, 1.75)	1.44 (1.19, 1.74)
Onset-address analysis d				
Total (≤ 100 km)	479,372	1.50 (1.33, 1.68)	1.52 (1.31, 1.77)	1.45 (1.20, 1.76)
≤ 50 km	461,069	1.49 (1.32, 1.68)	1.53 (1.32, 1.79)	1.42 (1.17, 1.72)
≤ 20 km	227,055	1.43 (1.20, 1.69)	1.49 (1.20, 1.86)	1.38 (1.09, 1.73)

Note:

a Extremely low temperature is the 1^st^ percentile of temperature, and the referent temperature is the minimum risk temperature.

b The main results were from models without adjustment for pollutants.

c The hospital-address analysis was conducted among participants with both hospital address and onset address. Meteorological data were retrieved from fixed-site monitors based on hospital addresses within 100 km to the nearest fixed-site monitoring station.

d The onset-address analysis was conducted among participants with both hospital address and onset address. Meteorological data were retrieved from fixed-site monitors based on onset addresses within 100, 50, 20 km to the nearest fixed-site monitoring station, respectively. Abbreviations: CIs, confidence intervals; AMI, acute myocardial infarction; STEMI, ST-segment-elevation myocardial infarction; NSTEMI, non-ST-segment-elevation myocardial infarction; PM_2.5_, fine particulate matter; NO_2_, nitrogen dioxide; SO_2_, sulfur dioxide; O_3_, ozone; CO, carbon monoxide; km, kilometer.

### Attributable fractions

Table 3 presents AFs of AMI associated with non-optimum temperatures at both national and regional levels. Given the overall non-significant effects of high temperature after accounting for the harvesting effects, we did not evaluate the heat effects in calculating disease burden attributable to non-optimum temperatures. We also did not calculate the disease burden during centralized-heating period due to the lack of effects associated with low temperature. Totally, there were 13.26% (95%eCI: 7.99%–18.01%) of AMI cases attributable to non-optimum temperatures nationally, with much smaller AF in the heating region (6.04%, 95%eCI: 2.42%–9.42%) than in the non-heating region (21.09%, 95%eCI: 14.03%–27.31%). In the non-heating region, the AF of AMI during winter (29.64%, 95%eCI: 16.04%–40.95%) were much higher than that in non-winter period (17.89%, 95%eCI: 13.28%–22.20%).

**Table 3:** Attributable fractions (%, means and 95%eCIs) of AMI due to non-optimum temperature.

Region	AFs (95%eCIs)
National	13.26 (7.99, 18.01)
Region with centralized heating	
Whole year	6.04 (2.42, 9.42)
Heating period	-
Non-heating period	9.36 (3.75, 14.61)
Region without centralized heating	
Whole year	21.09 (14.03, 27.31)
Winter	29.64 (16.04, 40.95)
Non-winter period	17.89 (13.28, 22.20)

Abbreviations: eCIs, empirical confidence intervals; AMI, acute myocardial infarction; AFs, attributable fractions.

## Discussion

To our knowledge, this nationwide analysis in 324 Chinese cities is the largest study to investigate the effects of ambient temperature on hourly AMI onset at the individual level, and the first study to assess burden of AMI onset attributable to non-optimum temperature. Almost monotonically increasing risks were observed for both overall AMI and its two subtypes when ambient temperature declined. The excess risks of AMI onset were observed during the whole year in the non-heating region and non-heating period in the heating region, but not during heating period. We did not observe significant effects of high temperature after accounting for the harvesting effects. Stronger effects were found among females and patients aged over 65. Further, taking spatial and seasonal heterogeneity into consideration, we made a more accurate evaluation on the burden of AMI onset due to non-optimum temperature. Much lower AFs were derived from the heating region (especially during heating period) than the non-heating region, implying a protective role of centralized heating in reducing disease burden due to non-optimum temperature.

The non-optimum temperature has been suggested to be a risk factor of AMI mortality and/or morbidity in previous studies.^13^^,^^16^^,^^18^ However, most previous studies examined the temperature-AMI relationship by fitting daily deaths/cases of AMI-related events and ambient temperature through time-series analyses.^16^^,^^18^ Consequently, their findings have always been challenged by the ecological fallacy and unclear chronological order of exposure and events within the same day. Though there were a limited number of case-crossover studies, few had utilized information of AMI onset at a sub-daily timescale.^20^^,^^23^^,^^26^ Thus, the critical advantage and novelty of our study lie in the careful analysis based on temperature exposures before the specific hour of AMI symptom onset at the individual level from a nationwide registry. We thus provide novel and reliable evidence on the adverse effects of non-optimum ambient temperature on AMI onset, which varied substantially across different regions and periods. Almost linear exposure–response curves over 0–21 days were observed among all regions except in the heating region during heating period. The plateauing and even decreasing phenomenon at low temperatures of the national curve might be driven by the null or weak effects of low temperature in heating region.

The associations between non-optimum temperatures and increased risk of AMI onset were biologically plausible. Both controlled human exposure studies and animal experiments demonstrated that low temperature could affect the autonomic nervous system, and subsequently induce peripheral vasoconstriction, as well as the elevation of arterial pressure and heart rate.^27^, ^28^, ^29^ All of these changes may increase cardiac workload and decrease coronary blood flow. Higher platelet viscosity and elevated plasma fibrinogen concentrations during cold exposure are also possible pathways to promote thrombosis.^30^^,^^31^ Moreover, there was evidence that cold temperature could induce coronary artery spasm, then exacerbate unstable angina and result in progression to AMI.^32^ The above mechanisms constitute the biological basis of the association between low ambient temperature and increased risk of AMI onset. The adverse cardiovascular effects of heat are also of some biological plausibility. High temperature may trigger AMI events through thermoregulation dysfunction and excess loss of body fluid, which could lead to increases in blood viscosity, cholesterol levels, platelet count, and hemoconcentration.^33^^,^^34^ Nevertheless, our analysis using hourly lags indicated that the heat effects were very modest and transient, and became non-significant after accounting for the harvesting effects. Similarly, a nationwide case-crossover study in United Kingdom observed a very transient effect of high temperature on myocardial infarction incidence that occurred only within 6 h after exposure, but the overall risks were still not significant.^23^ Different lag patterns of high and low temperatures underlined that prompt and timely protections would be needed to reduce heat-related AMI onset, while prolonged preventive measures could help address cold-related risks.^18^ Further investigations are warranted to clarify the heat effects on AMI onset and their time course.

Stratified analyses illustrated higher risk of AMI onset associated with cold temperature among individuals aged 65 years or older, which was consistent with several previous studies.^15^^,^^16^^,^^18^^,^^35^ Possible reasons may be related to the vulnerable cardiovascular system and impaired thermoregulation function among elderly people, leading to greater increases in myocardial oxygen demand during cold exposure.^36^ Moreover, the pre-existing comorbidities among the elderly might further contribute to their susceptibility to cold.^35^ Consistent with previous evidence,^18^^,^^37^^,^^38^ we observed larger effect estimates among females. This might be partly explained by the lower mass of muscle (an organ of heat production) in females than males so that females have more difficulty maintaining their body temperature due to additional distribution of blood flow to the uteri and ovaries.^37^ However, another two studies reported higher risk of AMI among males than females associated with low temperatures.^15^^,^^39^ The discrepancy could be attributable to the differences in study design, population characteristics, and sample size, and thus more studies are still needed to clarify this issue.

We estimated that 13.26% of the total AMI cases across 324 Chinese cities were attributable to non-optimum temperature. To our knowledge, there has been no study quantifying the burden of AMI onset attributable to non-optimum temperatures. Therefore, it is impossible to compare our results with previous estimates. Still, the AF from our data was generally comparable to the AF of IHD deaths for China from the GBD Study 2019, which estimated a significant burden (AF: 8.08%) for non-optimum temperature.^4^ However, GBD Study 2019 failed to adequately address the complexities of temperature–mortality relationships (i.e., the lagged effects of temperature).^5^ Further, a multi-city time-series study in China reported larger fractions of mortality attributable to non-optimum temperatures (i.e., 18.8% for coronary heart disease).^18^ The heterogeneity might be due to differences in health outcome (mortality vs. morbidity), study design, and whether using spatial and seasonal exposure–response relationships. By virtue of the individual-level case-crossover design with hourly onset information, we provided more reliable and accurate estimation on the relative risk and disease burden due to non-optimum temperature.

The present study illustrated the spatiotemporal heterogeneity of AMI risk and burden associated with non-optimum temperature across China. In non-heating region, we observed higher AF (29.64%) during winter than non-winter period (17.89%). The much larger AF in winter is reasonable because of the lower temperature, which was also reported in a multi-city time-series study.^7^ We additionally observed a much lower AF in heating region than in non-heating region, which might be due to a number of unmeasured differential factors (including the lifestyle, socio-economic status, climate pattern, and the long-term local acclimatization of residents) in the two regions. More importantly, the use of indoor heating system during cold seasons may dominate the difference in disease burden due to low temperature, because we did not observe any excess risks of AMI onset during heating period in the centralized-heating region. This study provides first-hand but preliminary evidence on the cardiovascular benefits of centralized heating against cold temperature. Given the relatively high prevalence of AMI in Chinese population, even a small decrease of AF could lead to considerable reductions in AMI cases.^16^^,^^40^ It can be reasonably speculated that expanding the geographical coverage or extending the duration of centralized heating may have the potential to reduce the burden of cardiovascular diseases. New heating technology using clean energy is also urgently needed under the changing climate. Still, given the uncontrolled residual confounding, more studies are warranted to further elucidate and corroborate the benefits of centralized heating.

Our findings provide different implications for various stakeholders. For policymakers, the varying temperature-AMI associations imply the need to design tailored public interventions and resilient infrastructures for specific regions and periods. Hospitals and medical staff should make sufficient preparedness preceding the extreme weather days. Patients, especially females and elders, should increase vigilance, reduce unnecessary outdoor activities, and use heating equipment or air conditioner if needed during extreme weather days to protect themselves.^41^^,^^42^ Finally, community education is also vitally needed to increase awareness of the public about risk factors of AMI and promote health behaviors to cope with the impact of extreme weather events.^7^

This study has several notable strengths. First, our data is collected from a standardized, nationwide AMI registry database covering all major cities and thus has good representativeness in Chinese mainland. The large sample size (1.05 million) also guaranteed sufficient statistical power. Second, the use of hourly information on symptom onset in analysis allowed us to examine the associations between ambient temperature and AMI onset with clear chronological order of exposure and events within the same day. Third, our results also provided novel and robust evidence on the AMI burden attributable to non-optimum temperatures, which may be helpful for further updates of GBD estimations. Last, we characterized the possible effect heterogeneity by regions and periods, which allowed an initial evaluation of potential effect modifications by the policy of centralized heating.

There are also several limitations. First, similar to previous epidemiological studies, we matched ambient temperature measured from nearby fixed-site monitors as a proxy of the individual exposure without accounting for temperature exposures in indoor environments, which could inevitably result in exposure misclassification. We did, however, conducted a sensitivity analysis by using temperature monitored at nearer fixed-site stations and observed similar results. Besides, such misclassification was likely to be random and could only bias our estimates towards the null.^43^ Second, the exposure data was matched to the hospital address rather than the location of AMI onset. Nevertheless, this resultant measurement error may not substantially affect our results in that most AMI patients were sent to the nearest hospital and the spatial variation of temperature within a city was generally limited. Furthermore, our sensitivity analyses in a subgroup also showed comparable estimates when using temperature matched based on the location of symptom onset. Third, the information on centralized heating was collected on the city level and therefore did not necessarily reflect actual use of centralized heating for each patient. Fourth, the time of ACS onset was self-reported, and could be subject to recall bias. However, we believe that such bias would not substantially influence our results due to its random nature in this nationwide database and the long lag period evaluated (up to 21 days). Fifth, we only included AMI patients admitted to hospitals, and those who died before arriving at the hospital were not captured. Sixth, some time-varying risk factors (e.g., lifestyle-related) could not be completely excluded, but the residual confounding may have limited impact on our findings because these variables are unlikely to vary substantially within one month. Last, we did not exactly know whether a registered patient is a recurrent AMI due to the de-identification nature of the present study, which could not influence our overall results but limited our ability in conducting a subgroup analysis. Still, this would not be a major concern because no consensus has reached on whether recurring AMI was more sensitive to a change of ambient temperature than incident AMI.^44^^,^^45^

In summary, in this nationwide case-crossover study of 1.05 million AMI patients from 324 Chinese cities, we found that non-optimum ambient temperature could significantly trigger AMI onset. For the first time we estimated the disease burden of AMI due to non-optimum temperature after accounting for spatial and seasonal heterogeneity of its effects. This study also provides novel evidence on the differential impact of non-optimum temperatures on AMI risk and burden by regions and periods, and highlights the potential protective role of centralized heating. Our findings may further facilitate public health strategies to alleviate the detrimental effects of non-optimum ambient temperature under global climate change.

## Contributors

YJ, JH, JG, YH and RC conceived the study. YJ and JH wrote the first and successive drafts of the manuscript. YJ and JH analyzed the data. LP, WF, HY, JC, WW, DX, XS, BY, YW, YX, LW, CL, YC, DZ, JG, YH, and RC carried out the programming to extract the data and curate the dataset. HL, JJ, HK, JG, YH, and RC edited and revised manuscript. JG, YH and RC verified the underlying data. All authors read, discussed, and approved the final version of the manuscript. The corresponding authors attests that all listed authors meet the authorship criteria and that no others meeting the criteria have been omitted, had full access to all data in the study, and had the final responsibility to submit it for publication.

## Data sharing statement

Anonymized data will be available through a formal application process which will be reviewed by the Data Management Committee of the CCA Database - Chest Pain Center.

## Declaration of interests

All authors declare no competing interests.

## Appendix Supplementary materials

Image, application 1
