# Supplementary material for: Non-optimum temperature increases risk and burden of acute myocardial infarction onset: A nationwide case-crossover study at hourly level in 324 Chinese cities
Source: eClinicalMedicine. 2022 Jun 17;50:101501. doi: 10.1016/j.eclinm.2022.101501 (PMC9218136; doi:10.1016/j.eclinm.2022.101501)
Supplement: Supplementary file 1 [file mmc1.pdf]

Supplementary Information

Non-optimum temperature increases risk and burden of acute myocardial infarction onset: a nationwide case-crossover study at hourly level in 324 Chinese cities

Yixuan Jiang, Jialu Hu, Li Peng, Huichu Li, John S. Ji, Weiyi Fang, Hongbing Yan, Jiyan Chen, Weimin Wang, Dingcheng Xiang, Xi Su, Bo Yu, Yan Wang, Yawei Xu, Lefeng Wang, Chunjie Li, Yundai Chen, Dong Zhao, Haidong Kan, Junbo Ge, Yong Huo, Renjie Chen

Supplemental Methods

Statistical analysis

Conditional logistic regression models combined with the distributed lag non-linear model (DLNM) were used to quantify the associations between ambient temperature and the onset of acute myocardial infarction (AMI) and its subtypes. The main model can be presented by the following formula:

$$\text{logit}\big(P(\text{case} = 1 \text{ in stratum } i)\big) = \alpha_{stratum\ i} + \beta\ DLNM(temperature) + \gamma\ \ ns(\text{RH}, 3) + \delta\ \text{Holiday} \quad (1)$$

In equation (1), a stratum consists of one case (case = 1) and its controls (case = 0), and the total number of strata is equal to the number of included patients. $P(\text{case} = 1 \text{ in stratum } i)$ is the conditional probability of being a case in the *i*th stratum. $\alpha_{stratum\ i}$ represents the constant or intercept of stratum *i* (each stratum has an intercept). $DLNM(temperature)$ is a matrix produced by a cross-basis function for temperature modeled by DLNM using natural cubic splines for both the exposure–response relationships and lag structures. A natural cubic spline function (degrees of freedom, *df*= 4) with three internal knots at equally spaced percentiles (P25, P50 and P75) of temperature ranges was fitted to account for potential non-linear exposure–response relationships. A natural cubic spline function (*df*= 4) with two internal knots at equally spaced log-values of lags (0–21 d) was applied to allow for more flexibility at shorter delays. $ns(\text{RH}, 3)$ is the natural cubic spline function of 0–21 days averaged relative humidity with 3 *df*; and Holiday is a binary variable indicating whether the date was a public holiday.

Supplemental Figures

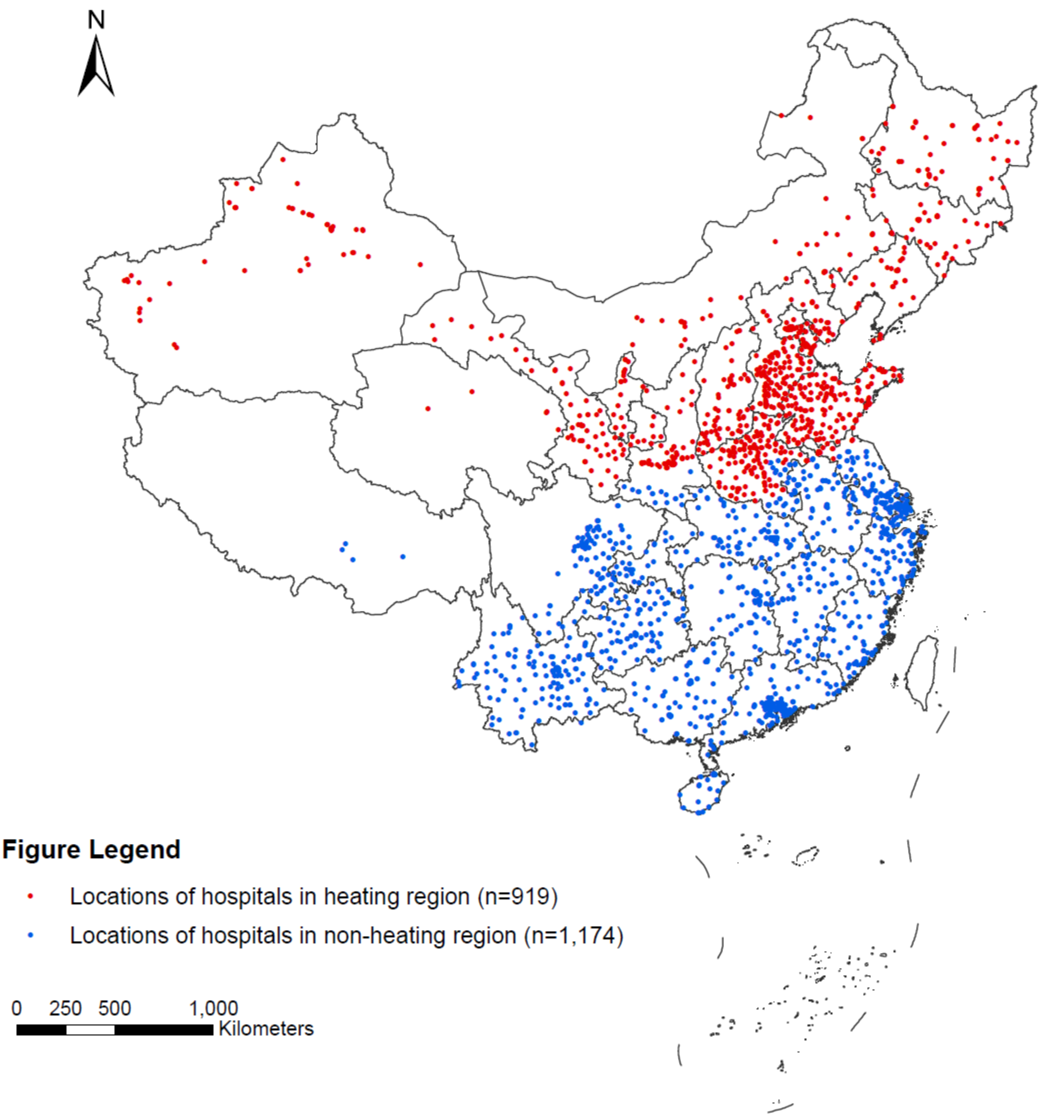

Figure S1. Geographic distribution of the 2,093 hospitals across China from 2015 to 2021.

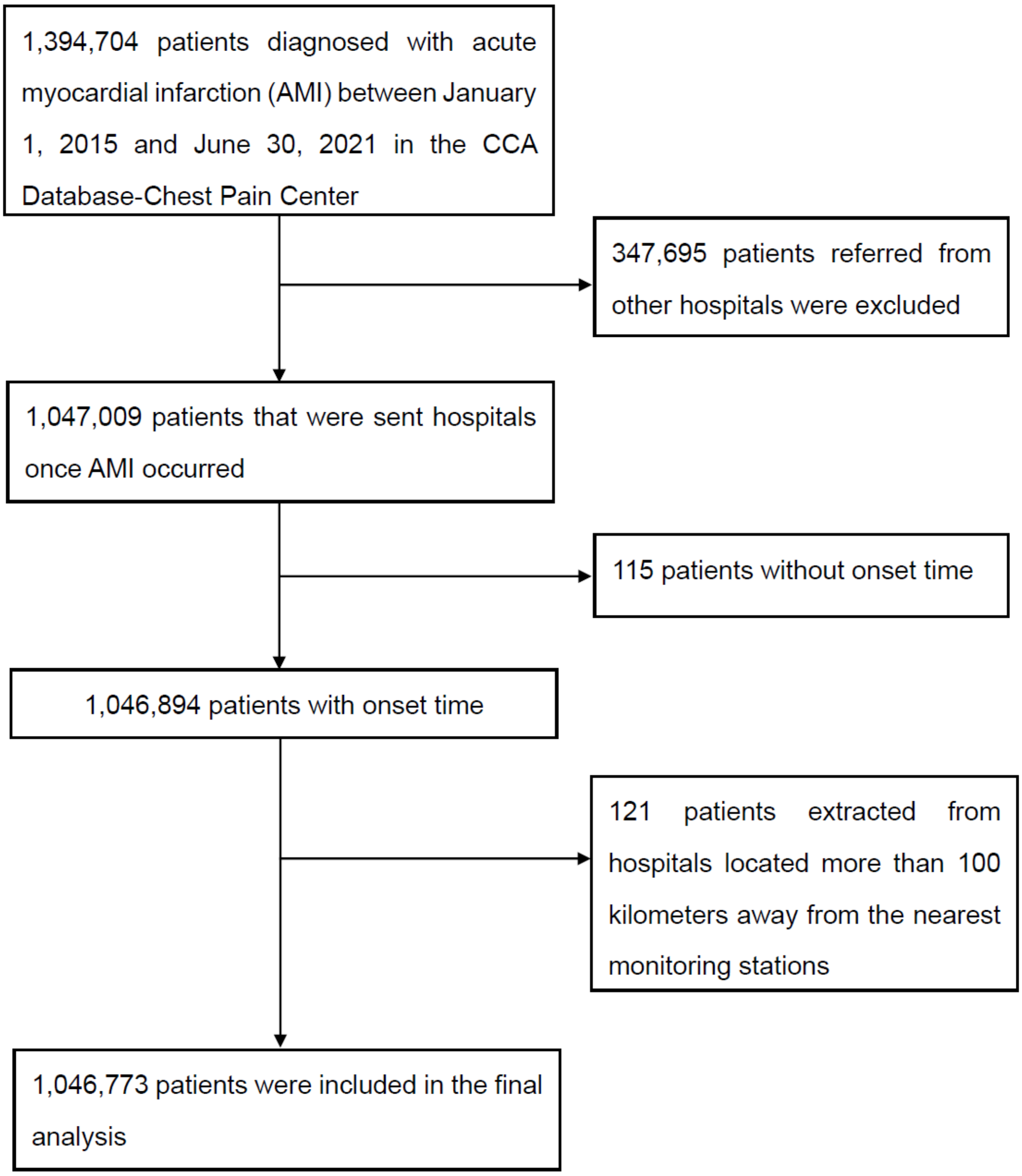

Figure S2. Flow chart of the study inclusion.

Abbreviation: AMI, acute myocardial infarction; CCA, Chinese Cardiovascular Association.

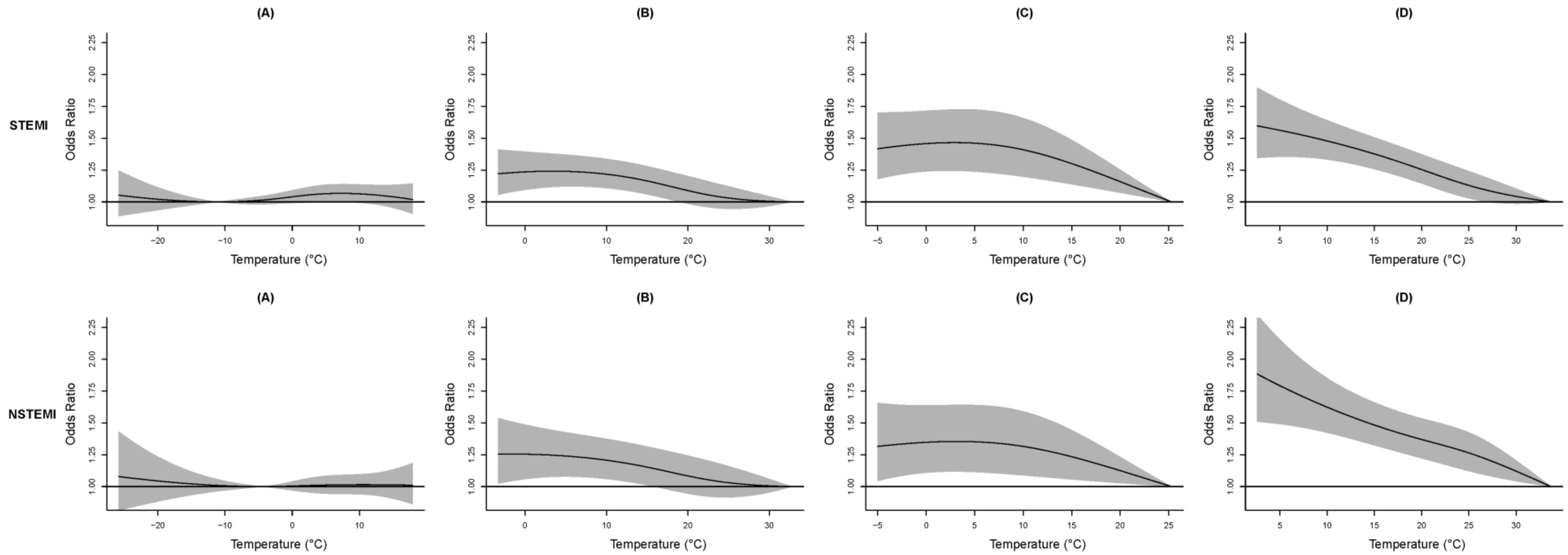

Figure S3. Cumulative exposure–response curves for associations of temperature with STEMI and NSTEMI over 0–21 days by region and period.

(A) heating region during heating period; (B) heating region during non-heating period; (C) non-heating region during winter; (D) non-heating region during non-winter period. The solid black lines are the odds ratios of AMI onset, and the gray areas are the 95% confidence intervals.

Abbreviations: STEMI, ST-segment-elevation myocardial infarction; NSTEMI, non-ST-segment-elevation myocardial infarction; AMI, acute myocardial infarction.

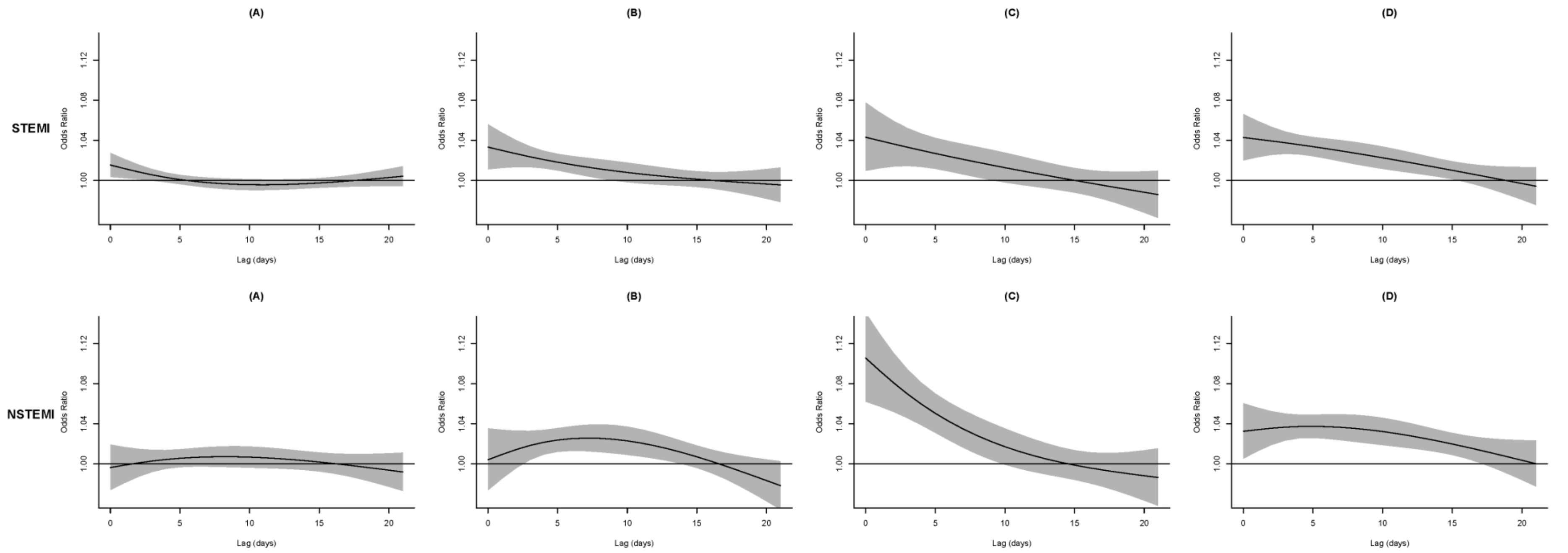

Figure S4. Lag structures for the associations of STEMI and NSTEMI with extremely low temperature in regions with centralized heating during heating period (A) and non-heating period (B), and in regions without centralized heating during winter (C) and non-winter period (D).

The solid black lines are the odds ratios (extremely low temperature: 1st percentile of temperature vs. the minimum risk temperature) of AMI onset, and the gray areas are the 95% confidence intervals.

Abbreviations: STEMI, ST-segment-elevation myocardial infarction; NSTEMI, non-ST-segment-elevation myocardial infarction; AMI, acute myocardial infarction.

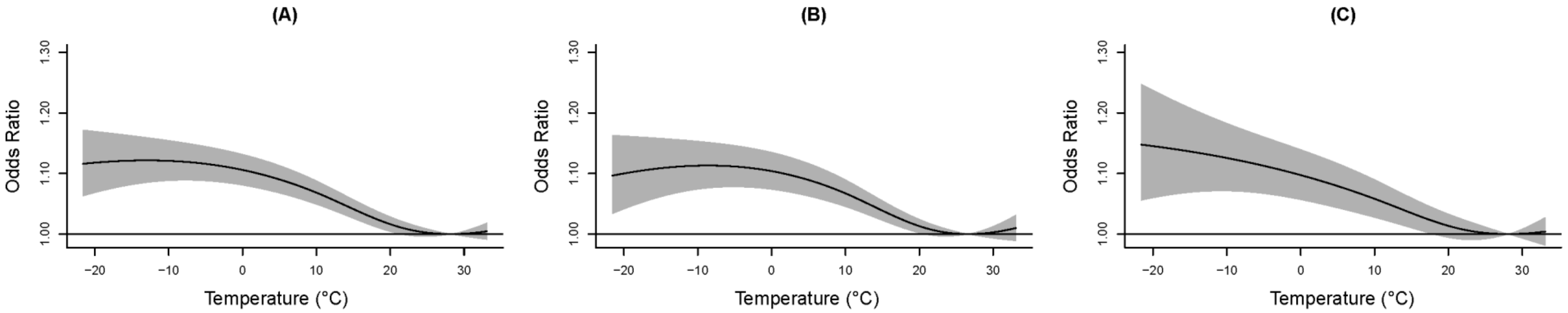

Figure S5. Cumulative exposure–response curves for the associations of temperature with AMI (A), STEMI (B) and NSTEMI (C) onset across 324 cities over 0–72 h.

The solid black lines are the odds ratios of AMI onset, and the gray areas are the 95% confidence intervals.

Abbreviations: AMI, acute myocardial infarction; STEMI, ST-segment-elevation myocardial infarction; NSTEMI, non-ST-segment-elevation myocardial infarction.

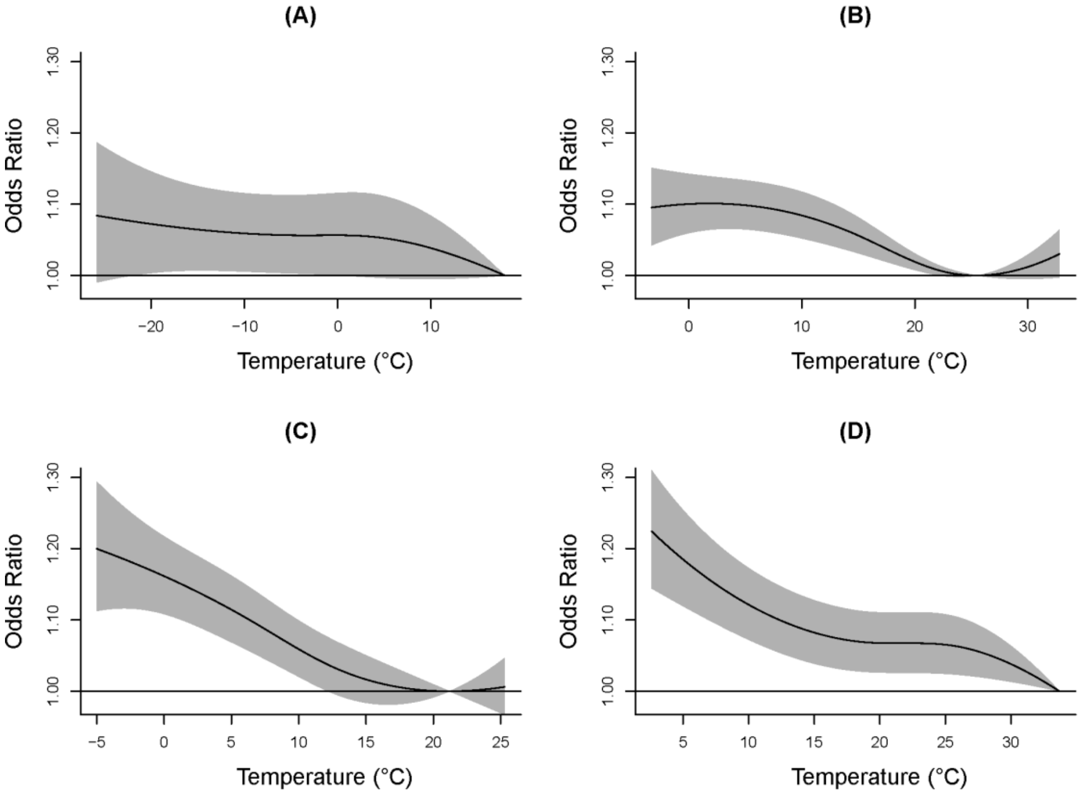

Figure S6. Cumulative exposure–response curves for temperature with AMI onset over 0–72 h by region and period.

(A) heating region during heating period; (B) heating region during non-heating period; (C) non-heating region during winter; (D) non-heating region during non-winter period. The solid black lines are the odds ratios of AMI onset, and the gray areas are the 95% confidence intervals.

Abbreviations: AMI, acute myocardial infarction.

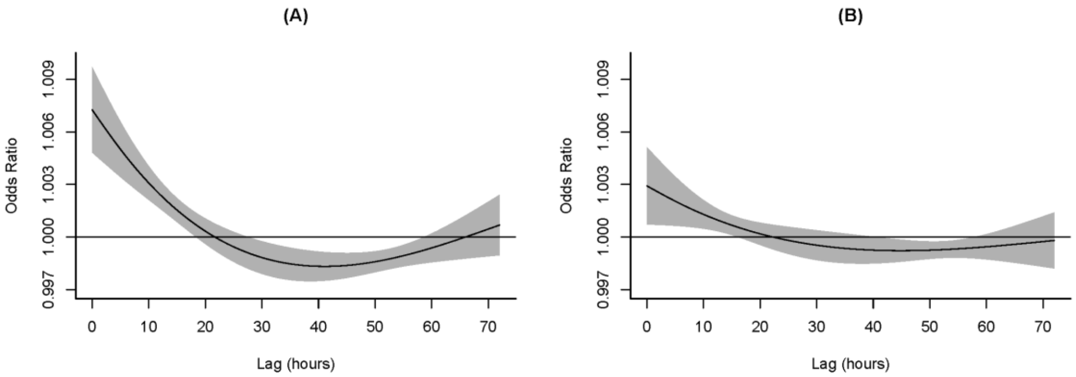

Figure S7. Lag structures for the associations of AMI onset with extremely high temperature by region and period.

(A) heating region during non-heating period; (B) non-heating region during winter. The solid black lines are the odds ratios (extremely high temperature: 99th percentile of temperature vs. the minimum risk temperature) of AMI onset, and the gray areas are the 95% confidence intervals.

Abbreviations: AMI, acute myocardial infarction.

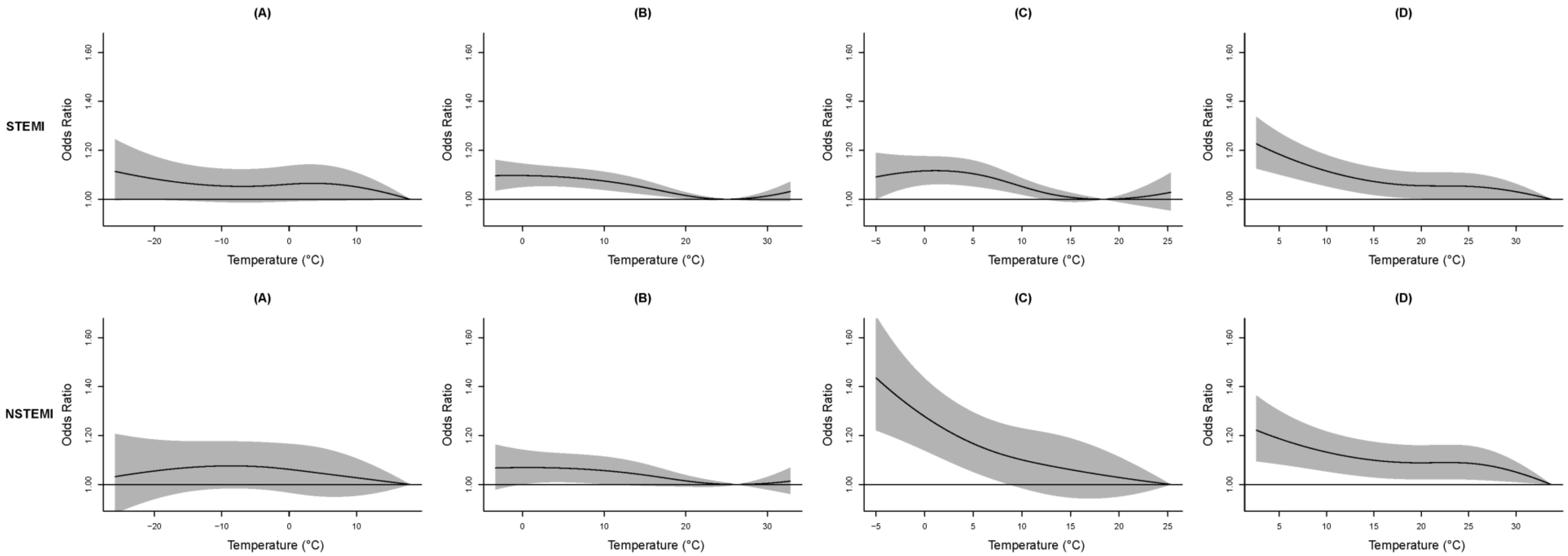

Figure S8. Cumulative exposure–response curves for associations of temperature with STEMI and NSTEMI over 0–72 h by region and period.

(A) heating region during heating period; (B) heating region during non-heating period; (C) non-heating region during winter; (D) non-heating region during non-winter period. The solid black lines are the odds ratios of AMI onset, and the gray areas are the 95% confidence intervals.

Abbreviations: STEMI, ST-segment-elevation myocardial infarction; NSTEMI, non-ST-segment-elevation myocardial infarction; AMI, acute myocardial infarction.

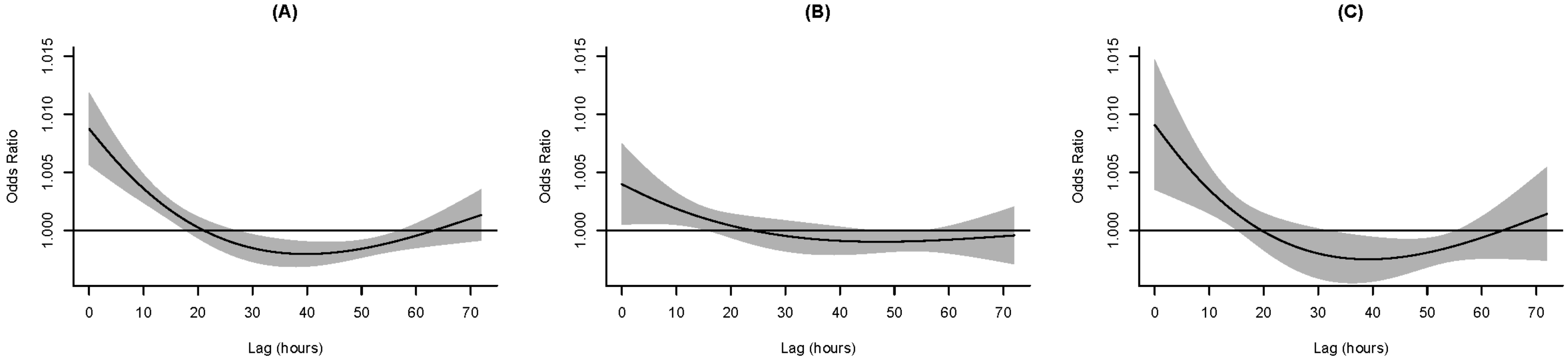

Figure S9. Lag structures for the associations of STEMI (A, C) and NSTEMI (B) onset with extremely high temperature by region and period.

(A, B) the heating region during non-heating period; (C) the non-heating region during winter. The solid black lines are the odds ratios (extremely high temperature: 99th percentile of temperature vs. the minimum risk temperature) of AMI onset, and the gray areas are the 95% confidence intervals.

Abbreviations: STEMI, ST-segment-elevation myocardial infarction; NSTEMI, non-ST-segment-elevation myocardial infarction; AMI, acute myocardial infarction.

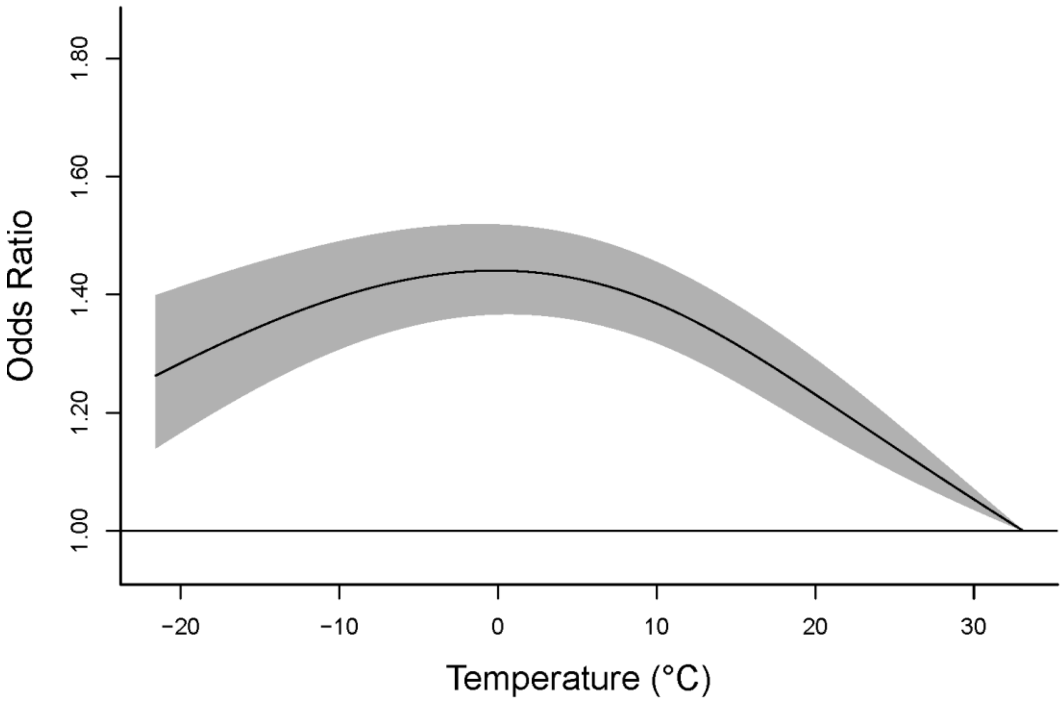

Figure S10. Cumulative exposure–response curves for associations of temperature with AMI across 324 cities over 0–21 days based on regular daily temperature.

Abbreviation: AMI, acute myocardial infarction.

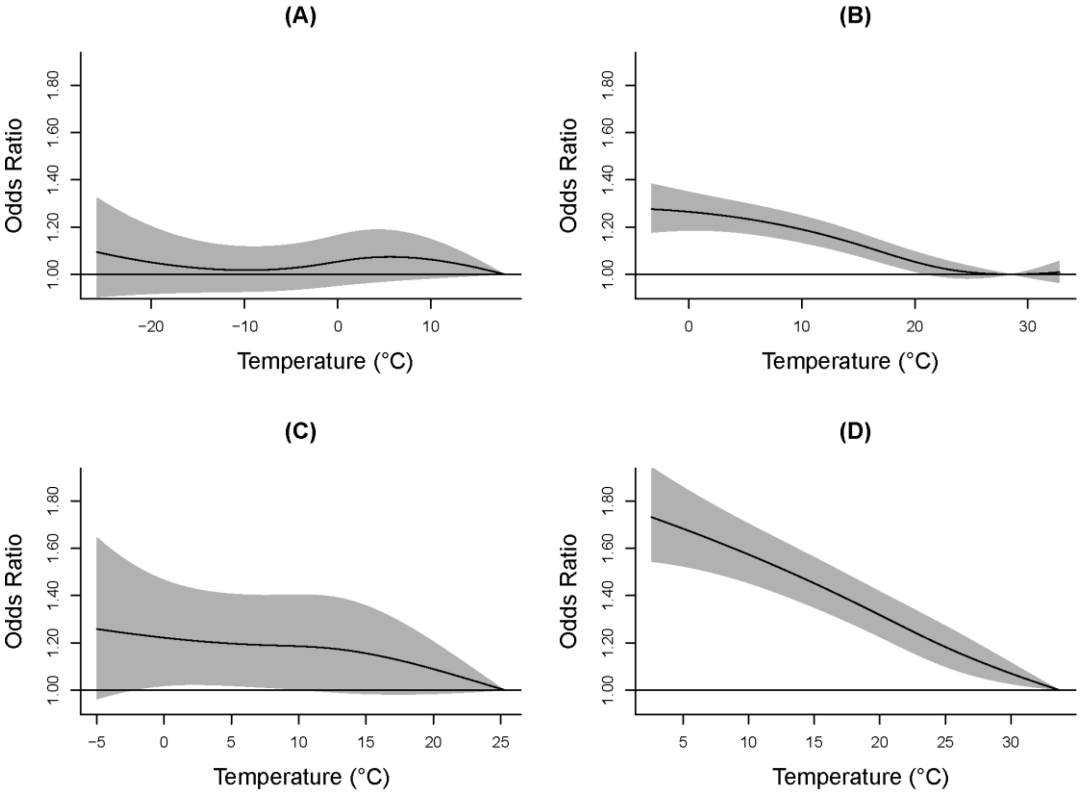

Figure S11. Cumulative exposure–response curves for associations of temperature with AMI across over 0–21 days by region and period based on regular daily temperature.

(A) heating region during heating period; (B) heating region during non-heating period; (C) non-heating region during winter; (D) non-heating region during non-winter period. The solid black lines are the odds ratios of AMI onset, and the gray areas are the 95% confidence intervals.

Abbreviation: AMI, acute myocardial infarction.

Supplemental Tables

Table S1. Demographic characteristics of all AMI cases in the CCA database-Chest Pain Center and cases included and excluded.

Demographic characteristics	All (n= 1,394,704)	Included (n= 1,046,773)	Excluded (n= 347,931)
Age at onset			
< 65	718,981 (51.6)	532,156 (50.8)	186,926 (53.7)
≥ 65	675,379 (48.4)	514,400 (49.1)	160,878 (46.2)
Missing	344 (0.0)	217 (0.0)	127 (0.0)
Sex			
Male	1,031,433 (74.0)	768,224 (73.4)	263,209 (75.6)
Female	363,206 (26.0)	278,503 (26.6)	84,703 (24.3)
Missing	65 (0.0)	46 (0.0)	19 (0.0)

Abbreviations: AMI, acute myocardial infarction; CCA, Chinese Cardiovascular Association.

Note: Values are n (%).

Table S2. Baseline characteristics of the study population.

**Baseline characteristics**	N, or N (%)
Case day	1,046,773
Control day	3,557,266
Age (year)	
< 65	532,156 (50.8)
≥ 65	514,400 (49.1)
Missing	217 (0.0)
Sex	
Male	768,224 (73.4)
Female	278,503 (26.6)
Missing	46 (0.0)
Disease type	
STEMI	663,929 (63.4)
NSTEMI	382,844 (36.6)
Centralized heating	
Yes	544,271 (52.0)
No	502,502 (48.0)

Abbreviations: STEMI, ST-segment-elevation myocardial infarction; NSTEMI, non-ST-segment-elevation myocardial infarction.

Table S3. Cumulative odds ratios (95%CIs) of overall AMI and AMI subtypes onset associated with extremely high temperature [a] by region and period.

Region	Extremely high temperature (°C)	Referent temperature (°C)	AMI	STEMI	NSTEMI
National	31.3	28.2	1.00 (0.99, 1.01)	1.01 (0.99, 1.02)	1.00 (0.99, 1.02)
HH	-	-	-	-	-
HNH	31.2	25.5	1.02 (0.99, 1.04)	1.02 (0.99, 1.05)	1.01 (0.97, 1.05)
NW	23.5	21.2	1.00 (0.98, 1.02)	1.02 (0.97, 1.07)	-
NNW	-	-	-	-	-

Note: [a] extremely high temperature refers to the 99th percentile of temperature, and the referent temperature is the minimum risk temperature.

Abbreviations: CIs, confidence intervals; AMI, acute myocardial infarction; STEMI, ST-segment-elevation myocardial infarction; NSTEMI, non-ST-segment-elevation myocardial infarction; HH, heating region during heating period; HNH, heating region during non-heating period; NW, non-heating region during winter; NNW, non-heating region during non-winter period.

Table S4. Cumulative odds ratios (95%CIs) of AMI onset associated with extremely low temperature [a] over 0–21 days by sex and age.

Subgroups	AMI	STEMI	NSTEMI
Sex			
Male	1.26 (1.16, 1.38) [b]	1.30 (1.16, 1.45)	1.19 (1.01, 1.39) [b]
Female	1.52 (1.31, 1.75) [b]	1.38 (1.15, 1.66)	1.77 (1.41, 2.21) [b]
Age			
< 65	1.25 (1.13, 1.39)	1.34 (1.18, 1.52)	1.10 (0.91, 1.32) [b]
≥ 65	1.41 (1.26, 1.57)	1.28 (1.11, 1.47)	1.66 (1.38, 1.99) [b]

Note: [a] extremely low temperature is the 1st percentile of temperature, and the referent temperature is the minimum risk temperature; [b] means significant between-subgroup difference.

Abbreviations: CIs, confidence intervals; AMI, acute myocardial infarction; STEMI, ST-segment-elevation myocardial infarction; NSTEMI, non-ST-segment-elevation myocardial infarction.

Table S5. Cumulative odds ratios (95%CIs) of AMI onset associated with extremely low temperature [a] based on regular daily temperature.

Region	OR (95%CI)
National	1.35 (1.26, 1.46)
HH	1.05 (0.92, 1.19)
HNH	1.26 (1.19, 1.34)
NW	1.24 (1.00, 1.52)
NNW	1.66 (1.51, 1.83)

Note: [a] extremely low temperature refers to the 1st percentile of temperature, and the referent temperature is the minimum risk temperature.

Abbreviations: CIs, confidence intervals; AMI, acute myocardial infarction; OR, odds ratio; HH, heating region during heating period; HNH, heating region during non-heating period; NW, non-heating region during winter; NNW, non-heating region during non-winter period.

Table S6. Cumulative odds ratios (95%CIs) of AMI onset associated with extremely low temperature [a] derived from models with different parameters.

df for temperature	*df* for lag	National	HH	HNH	NW	NNW
4	4	1.33 (1.24, 1.44)	1.02 (0.95, 1.10)	1.24 (1.13, 1.37)	1.46 (1.20, 1.76)	1.62 (1.46, 1.81)
	5	1.29 (1.20, 1.40)	1.02 (0.95, 1.09)	1.22 (1.13, 1.33)	1.38 (1.14, 1.68)	1.60 (1.44, 1.78)
	6	1.29 (1.20, 1.39)	1.01 (0.95, 1.09)	1.22 (1.13, 1.32)	1.38 (1.14, 1.68)	1.60 (1.44, 1.78)
	7	1.31 (1.22, 1.41)	1.02 (0.95, 1.10)	1.23 (1.13, 1.34)	1.44 (1.19, 1.75)	1.60 (1.44, 1.78)
5	4	1.32 (1.21, 1.43)	1.07 (0.93, 1.22)	1.24 (1.16, 1.32)	1.34 (1.07, 1.68)	1.61 (1.42, 1.84)
	5	1.28 (1.18, 1.39)	1.08 (0.92, 1.26)	1.23 (1.15, 1.31)	1.27 (1.04, 1.55)	1.59 (1.39, 1.81)
	6	1.28 (1.18, 1.39)	1.07 (0.92, 1.26)	1.23 (1.15, 1.31)	1.27 (1.05, 1.55)	1.59 (1.39, 1.81)
	7	1.30 (1.20, 1.42)	1.07 (0.94, 1.22)	1.23 (1.15, 1.32)	1.32 (1.09, 1.61)	1.59 (1.39, 1.81)
6	4	1.29 (1.20, 1.38)	1.07 (0.93, 1.23)	1.23 (1.15, 1.32)	1.36 (1.06, 1.73)	1.61 (1.40, 1.84)
	5	1.25 (1.17, 1.34)	1.08 (0.92, 1.28)	1.22 (1.14, 1.31)	1.29 (1.01, 1.65)	1.59 (1.38, 1.82)
	6	1.25 (1.17, 1.34)	1.08 (0.92, 1.28)	1.22 (1.14, 1.31)	1.30 (1.01, 1.66)	1.59 (1.39, 1.82)
	7	1.27 (1.18, 1.37)	1.07 (0.93, 1.24)	1.22 (1.14, 1.31)	1.33 (1.04, 1.70)	1.58 (1.38, 1.82)
7	4	1.31 (1.21, 1.42)	1.06 (0.92, 1.22)	1.24 (1.16, 1.32)	1.48 (1.22, 1.81)	1.61 (1.44, 1.80)
	5	1.28 (1.18, 1.38)	1.04 (0.90, 1.21)	1.23 (1.15, 1.31)	1.41 (1.15, 1.72)	1.58 (1.41, 1.77)

	6	1.27 (1.18, 1.38)	1.05 (0.90, 1.21)	1.23 (1.15, 1.31)	1.42 (1.16, 1.73)	1.58 (1.41, 1.77)
	7	1.29 (1.20, 1.40)	1.06 (0.92, 1.23)	1.23 (1.15, 1.31)	1.47 (1.20, 1.79)	1.58 (1.41, 1.77)

Note: [a] extremely low temperature refers to the 1st percentile of temperature, and the referent temperature is the minimum risk temperature.

Abbreviations: CIs, confidence intervals; AMI, acute myocardial infarction; HH, heating region during heating period; HNH, heating region during non-heating period; NW, non-heating region during winter; NNW, non-heating region during non-winter period.

Table S7. Cumulative odds ratios (95%CIs) of AMI onset associated with extremely low temperature [a] derived from models with different maximum of lag.

Lag period	Region	OR (95%CI)
0–21 d	National	1.33 (1.24, 1.44)
	HH	1.02 (0.95, 1.10)
	HNH	1.24 (1.13, 1.37)
	NW	1.46 (1.20, 1.76)
	NNW	1.62 (1.46, 1.81)
0–14 d	National	1.37 (1.28, 1.46)
	HH	1.02 (0.97, 1.08)
	HNH	1.27 (1.18, 1.38)
	NW	1.54 (1.32, 1.79)
	NNW	1.57 (1.44, 1.71)
0–28 d	National	1.31 (1.21, 1.41)
	HH	1.00 (0.95, 1.06)
	HNH	1.25 (1.13, 1.38)
	NW	1.48 (1.21, 1.82)
	NNW	1.62 (1.45, 1.80)

Note: [a] extremely low temperature refers to the 1st percentile of temperature, and the referent temperature is the minimum risk temperature.

Abbreviations: CIs, confidence intervals; AMI, acute myocardial infarction; OR, odds ratio; HH, heating region during heating period; HNH, heating region during non-heating period; NW, non-heating region during winter; NNW, non-heating region during non-winter period.

Table S8. The cumulative odds ratios (95%CIs) of AMI onset associated with extremely low temperature [a] derived from the main model and pooled by the meta-analysis.

Region	OR (95%CI)	
	Main model	Meta-analysis
National	1.33 (1.24, 1.44)	1.30 (1.25, 1.36)
HH	1.02 (0.95, 1.10)	1.00 (0.98, 1.03)
HNH	1.24 (1.13, 1.37)	1.23 (1.17, 1.29)
NW	1.46 (1.20, 1.76)	1.46 (1.23, 1.73)
NNW	1.62 (1.46, 1.81)	1.64 (1.47, 1.84)

Note: [a] extremely low temperature refers to the 1st percentile of temperature, and the referent temperature is the minimum risk temperature.

Abbreviations: CIs, confidence intervals; AMI, acute myocardial infarction; OR, odds ratio; HH, heating region during heating period; HNH, heating region during non-heating period; NW, non-heating region during winter; NNW, non-heating region during non-winter period.
